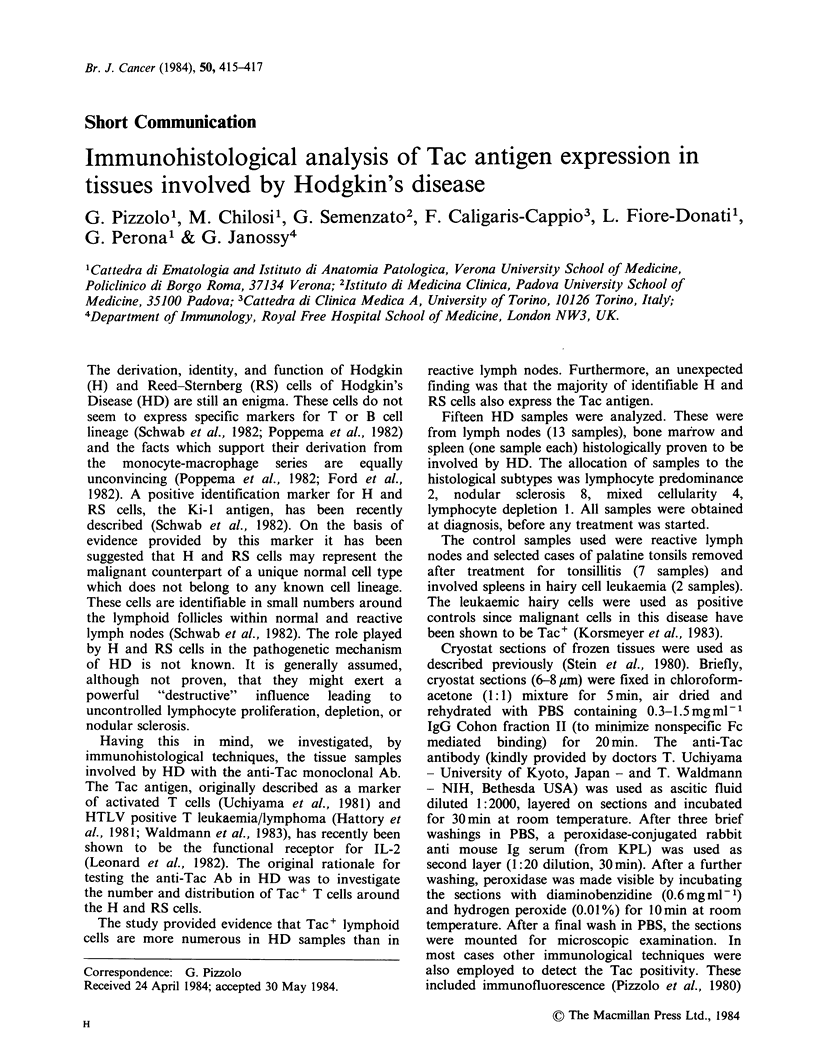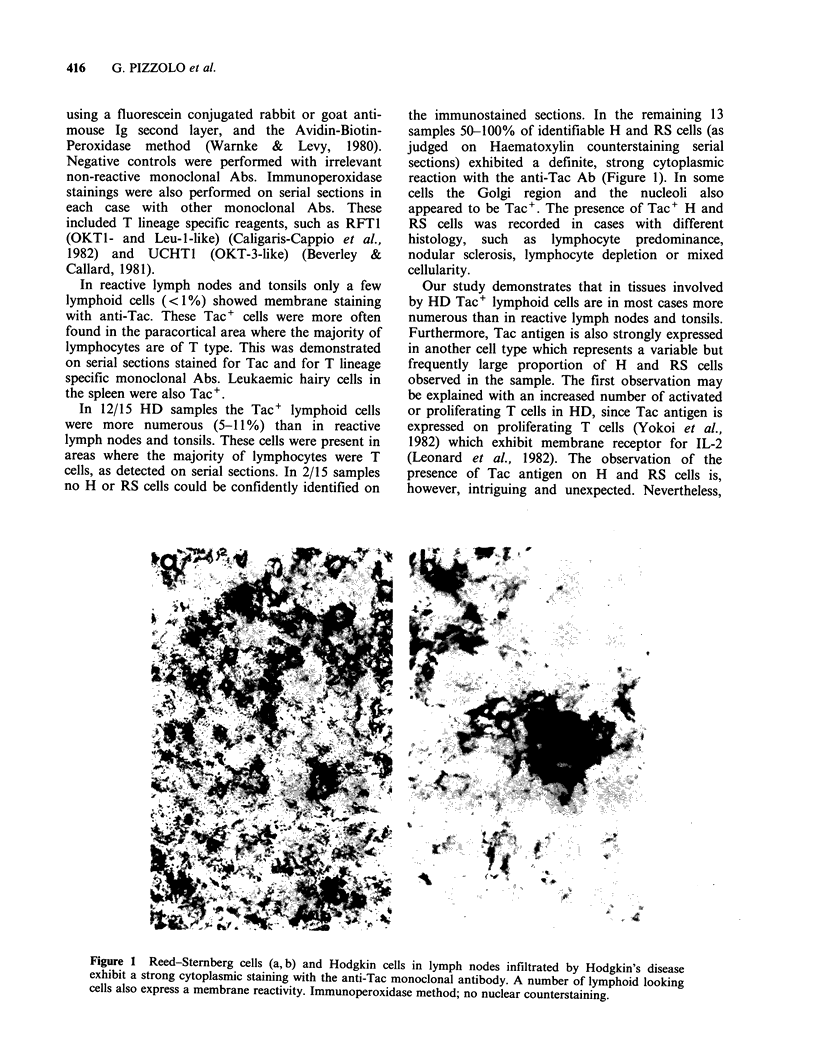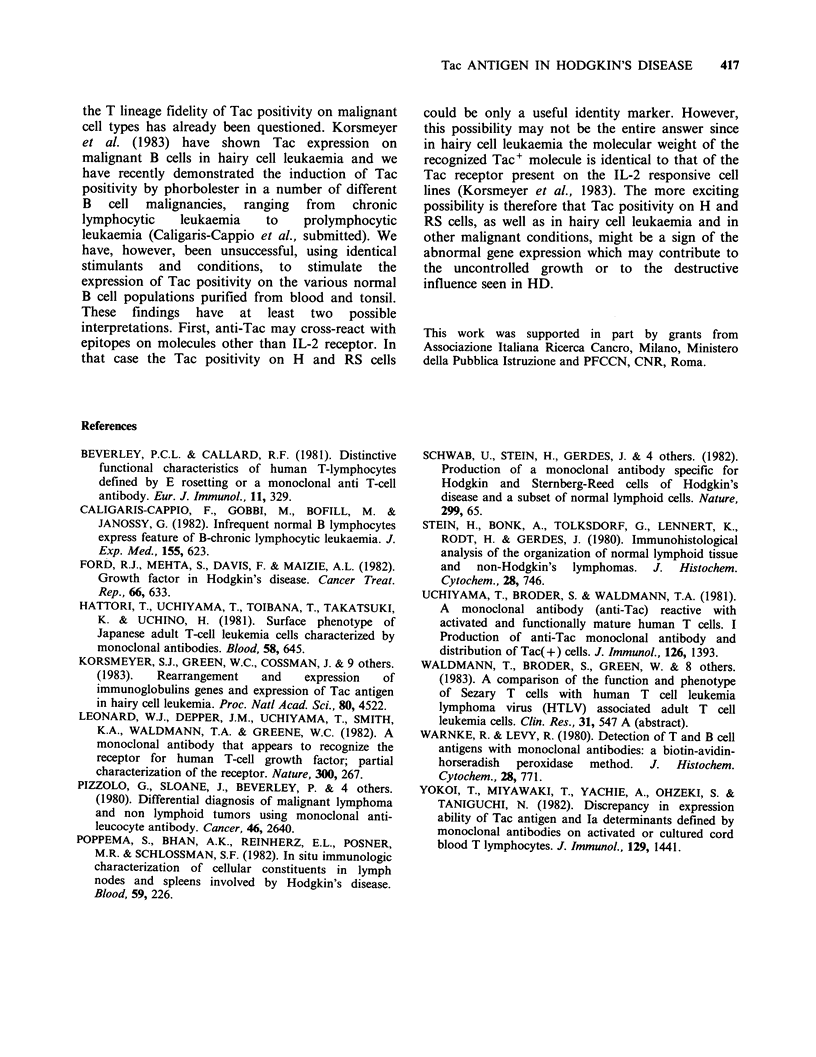# Immunohistological analysis of Tac antigen expression in tissues involved by Hodgkin's disease.

**DOI:** 10.1038/bjc.1984.191

**Published:** 1984-09

**Authors:** G. Pizzolo, M. Chilosi, G. Semenzato, F. Caligaris-Cappio, L. Fiore-Donati, G. Perona, G. Janossy

## Abstract

**Images:**


*Br. J. Cancer* (1984), **50**, 415–417

### Short Communication

### Immunohistological analysis of Tac antigen expression in tissues involved by Hodgkin's disease

G. Pizzolo[1], M. Chilosi[1], G. Semenzato[2], F. Caligaris-Cappio[3], L. Fiore-Donati[1], G. Perona[1] & G. Janossy[4]

[1]*Cattedra di Ematologia and Istituto di Anatomia Patologica, Verona University School of Medicine, Policlinico di Borgo Roma, 37134 Verona;* [2]*Istituto di Medicina Clinica, Padova University School of Medicine, 35100 Padova;* [3]*Cattedra di Clinica Medica A, University of Torino, 10126 Torino, Italy;* [4]*Department of Immunology, Royal Free Hospital School of Medicine, London NW3, UK.*

The derivation, identity, and function of Hodgkin (H) and Reed–Sternberg (RS) cells of Hodgkin's Disease (HD) are still an enigma. These cells do not seem to express specific markers for T or B cell lineage (Schwab *et al.*, 1982; Poppema *et al.*, 1982) and the facts which support their derivation from the monocyte-macrophage series are equally unconvincing (Poppema *et al.*, 1982; Ford *et al.*, 1982). A positive identification marker for H and RS cells, the Ki-1 antigen, has been recently described (Schwab *et al.*, 1982). On the basis of evidence provided by this marker it has been suggested that H and RS cells may represent the malignant counterpart of a unique normal cell type which does not belong to any known cell lineage. These cells are identifiable in small numbers around the lymphoid follicles within normal and reactive lymph nodes (Schwab *et al.*, 1982). The role played by H and RS cells in the pathogenetic mechanism of HD is not known. It is generally assumed, although not proven, that they might exert a powerful "destructive" influence leading to uncontrolled lymphocyte proliferation, depletion, or nodular sclerosis.

Having this in mind, we investigated, by immunohistological techniques, the tissue samples involved by HD with the anti-Tac monoclonal Ab. The Tac antigen, originally described as a marker of activated T cells (Uchiyama *et al.*, 1981) and HTLV positive T leukaemia/lymphoma (Hattory *et al.*, 1981; Waldmann *et al.*, 1983), has recently been shown to be the functional receptor for IL-2 (Leonard *et al.*, 1982). The original rationale for testing the anti-Tac Ab in HD was to investigate the number and distribution of $Tac^+$ T cells around the H and RS cells.

The study provided evidence that $Tac^+$ lymphoid cells are more numerous in HD samples than in reactive lymph nodes. Furthermore, an unexpected finding was that the majority of identifiable H and RS cells also express the Tac antigen.

Fifteen HD samples were analyzed. These were from lymph nodes (13 samples), bone marrow and spleen (one sample each) histologically proven to be involved by HD. The allocation of samples to the histological subtypes was lymphocyte predominance 2, nodular sclerosis 8, mixed cellularity 4, lymphocyte depletion 1. All samples were obtained at diagnosis, before any treatment was started.

The control samples used were reactive lymph nodes and selected cases of palatine tonsils removed after treatment for tonsillitis (7 samples) and involved spleens in hairy cell leukaemia (2 samples). The leukaemic hairy cells were used as positive controls since malignant cells in this disease have been shown to be $Tac^+$ (Korsmeyer *et al.*, 1983).

Cryostat sections of frozen tissues were used as described previously (Stein *et al.*, 1980). Briefly, cryostat sections (6–8 $\mu$m) were fixed in chloroform-acetone (1:1) mixture for 5 min, air dried and rehydrated with PBS containing 0.3–1.5 mg $ml^{-1}$ IgG Cohon fraction II (to minimize nonspecific Fc mediated binding) for 20 min. The anti-Tac antibody (kindly provided by doctors T. Uchiyama – University of Kyoto, Japan – and T. Waldmann – NIH, Bethesda USA) was used as ascitic fluid diluted 1:2000, layered on sections and incubated for 30 min at room temperature. After three brief washings in PBS, a peroxidase-conjugated rabbit anti mouse Ig serum (from KPL) was used as second layer (1:20 dilution, 30 min). After a further washing, peroxidase was made visible by incubating the sections with diaminobenzidine (0.6 mg $ml^{-1}$) and hydrogen peroxide (0.01%) for 10 min at room temperature. After a final wash in PBS, the sections were mounted for microscopic examination. In most cases other immunological techniques were also employed to detect the Tac positivity. These included immunofluorescence (Pizzolo *et al.*, 1980)

Correspondence: G. Pizzolo
Received 24 April 1984; accepted 30 May 1984.

© The Macmillan Press Ltd., 1984

using a fluorescein conjugated rabbit or goat anti-mouse Ig second layer, and the Avidin-Biotin-Peroxidase method (Warnke & Levy, 1980). Negative controls were performed with irrelevant non-reactive monoclonal Abs. Immunoperoxidase stainings were also performed on serial sections in each case with other monoclonal Abs. These included T lineage specific reagents, such as RFT1 (OKT1- and Leu-1-like) (Caligaris-Cappio *et al.*, 1982) and UCHT1 (OKT-3-like) (Beverley & Callard, 1981).

In reactive lymph nodes and tonsils only a few lymphoid cells (<1%) showed membrane staining with anti-Tac. These $Tac^+$ cells were more often found in the paracortical area where the majority of lymphocytes are of T type. This was demonstrated on serial sections stained for Tac and for T lineage specific monoclonal Abs. Leukaemic hairy cells in the spleen were also $Tac^+$.

In 12/15 HD samples the $Tac^+$ lymphoid cells were more numerous (5–11%) than in reactive lymph nodes and tonsils. These cells were present in areas where the majority of lymphocytes were T cells, as detected on serial sections. In 2/15 samples no H or RS cells could be confidently identified on the immunostained sections. In the remaining 13 samples 50–100% of identifiable H and RS cells (as judged on Haematoxylin counterstaining serial sections) exhibited a definite, strong cytoplasmic reaction with the anti-Tac Ab (Figure 1). In some cells the Golgi region and the nucleoli also appeared to be $Tac^+$. The presence of $Tac^+$ H and RS cells was recorded in cases with different histology, such as lymphocyte predominance, nodular sclerosis, lymphocyte depletion or mixed cellularity.

Our study demonstrates that in tissues involved by HD $Tac^+$ lymphoid cells are in most cases more numerous than in reactive lymph nodes and tonsils. Furthermore, Tac antigen is also strongly expressed in another cell type which represents a variable but frequently large proportion of H and RS cells observed in the sample. The first observation may be explained with an increased number of activated or proliferating T cells in HD, since Tac antigen is expressed on proliferating T cells (Yokoi *et al.*, 1982) which exhibit membrane receptor for IL-2 (Leonard *et al.*, 1982). The observation of the presence of Tac antigen on H and RS cells is, however, intriguing and unexpected. Nevertheless,

**Figure 1** Reed–Sternberg cells (a, b) and Hodgkin cells in lymph nodes infiltrated by Hodgkin's disease exhibit a strong cytoplasmic staining with the anti-Tac monoclonal antibody. A number of lymphoid looking cells also express a membrane reactivity. Immunoperoxidase method; no nuclear counterstaining.

the T lineage fidelity of Tac positivity on malignant cell types has already been questioned. Korsmeyer *et al.* (1983) have shown Tac expression on malignant B cells in hairy cell leukaemia and we have recently demonstrated the induction of Tac positivity by phorbolester in a number of different B cell malignancies, ranging from chronic lymphocytic leukaemia to prolymphocytic leukaemia (Caligaris-Cappio *et al.*, submitted). We have, however, been unsuccessful, using identical stimulants and conditions, to stimulate the expression of Tac positivity on the various normal B cell populations purified from blood and tonsil. These findings have at least two possible interpretations. First, anti-Tac may cross-react with epitopes on molecules other than IL-2 receptor. In that case the Tac positivity on H and RS cells could be only a useful identity marker. However, this possibility may not be the entire answer since in hairy cell leukaemia the molecular weight of the recognized $Tac^+$ molecule is identical to that of the Tac receptor present on the IL-2 responsive cell lines (Korsmeyer *et al.*, 1983). The more exciting possibility is therefore that Tac positivity on H and RS cells, as well as in hairy cell leukaemia and in other malignant conditions, might be a sign of the abnormal gene expression which may contribute to the uncontrolled growth or to the destructive influence seen in HD.

This work was supported in part by grants from Associazione Italiana Ricerca Cancro, Milano, Ministero della Pubblica Istruzione and PFCCN, CNR, Roma.

### References

BEVERLEY, P.C.L. & CALLARD, R.F. (1981). Distinctive functional characteristics of human T-lymphocytes defined by E rosetting or a monoclonal anti T-cell antibody. *Eur. J. Immunol.*, **11**, 329.

CALIGARIS-CAPPIO, F., GOBBI, M., BOFILL, M. & JANOSSY, G. (1982). Infrequent normal B lymphocytes express feature of B-chronic lymphocytic leukaemia. *J. Exp. Med.*, **155**, 623.

FORD, R.J., MEHTA, S., DAVIS, F. & MAIZIE, A.L. (1982). Growth factor in Hodgkin's disease. *Cancer Treat. Rep.*, **66**, 633.

HATTORI, T., UCHIYAMA, T., TOIBANA, T., TAKATSUKI, K. & UCHINO, H. (1981). Surface phenotype of Japanese adult T-cell leukemia cells characterized by monoclonal antibodies. *Blood*, **58**, 645.

KORSMEYER, S.J., GREEN, W.C., COSSMAN, J. & 9 others. (1983). Rearrangement and expression of immunoglobulins genes and expression of Tac antigen in hairy cell leukemia. *Proc. Natl Acad. Sci.*, **80**, 4522.

LEONARD, W.J., DEPPER, J.M., UCHIYAMA, T., SMITH, K.A., WALDMANN, T.A. & GREENE, W.C. (1982). A monoclonal antibody that appears to recognize the receptor for human T-cell growth factor; partial characterization of the receptor. *Nature*, **300**, 267.

PIZZOLO, G., SLOANE, J., BEVERLEY, P. & 4 others. (1980). Differential diagnosis of malignant lymphoma and non lymphoid tumors using monoclonal anti-leucocyte antibody. *Cancer*, **46**, 2640.

POPPEMA, S., BHAN, A.K., REINHERZ, E.L., POSNER, M.R. & SCHLOSSMAN, S.F. (1982). In situ immunologic characterization of cellular constituents in lymph nodes and spleens involved by Hodgkin's disease. *Blood*, **59**, 226.

SCHWAB, U., STEIN, H., GERDES, J. & 4 others. (1982). Production of a monoclonal antibody specific for Hodgkin and Sternberg-Reed cells of Hodgkin's disease and a subset of normal lymphoid cells. *Nature*, **299**, 65.

STEIN, H., BONK, A., TOLKSDORF, G., LENNERT, K., RODT, H. & GERDES, J. (1980). Immunohistological analysis of the organization of normal lymphoid tissue and non-Hodgkin's lymphomas. *J. Histochem. Cytochem.*, **28**, 746.

UCHIYAMA, T., BRODER, S. & WALDMANN, T.A. (1981). A monoclonal antibody (anti-Tac) reactive with activated and functionally mature human T cells. I Production of anti-Tac monoclonal antibody and distribution of Tac(+) cells. *J. Immunol.*, **126**, 1393.

WALDMANN, T., BRODER, S., GREEN, W. & 8 others. (1983). A comparison of the function and phenotype of Sezary T cells with human T cell leukemia lymphoma virus (HTLV) associated adult T cell leukemia cells. *Clin. Res.*, **31**, 547 A (abstract).

WARNKE, R. & LEVY, R. (1980). Detection of T and B cell antigens with monoclonal antibodies: a biotin-avidin-horseradish peroxidase method. *J. Histochem. Cytochem.*, **28**, 771.

YOKOI, T., MIYAWAKI, T., YACHIE, A., OHZEKI, S. & TANIGUCHI, N. (1982). Discrepancy in expression ability of Tac antigen and Ia determinants defined by monoclonal antibodies on activated or cultured cord blood T lymphocytes. *J. Immunol.*, **129**, 1441.